# Paritaprevir as a pan-antiviral against different flaviviruses

**DOI:** 10.3389/fmolb.2025.1524951

**Published:** 2025-04-03

**Authors:** R. P. Yadav, N. R. Jena

**Affiliations:** Discipline of Natural Sciences, Indian Institute of Information Technology, Design and Manufacturing, Jabalpur, India

**Keywords:** Zika virus, Dengue virus, West Nile virus, NS2B-NS3 protease, pan antiviral, docking, MD-simulations

## Abstract

**Introduction:**

The flavivirus infections caused by the Zika virus (ZIKV), Dengue virus (DENV), and West Nile virus (WNV) cause mild to serious pathological conditions, such as fever, joint pain, shock, internal bleeding, organ failure, nausea, breathlessness, brain tissue damage, neurodegenerative diseases, and deaths. As currently no efficient vaccine or drug is available to prevent or treat these diseases in humans, it is essential to identify potential drug-like molecules to treat these diseases. For these reasons, several known anti-viral drugs are repurposed against the proteases of ZIKV, WNV, and DENV to inhibit their activities.

**Methods:**

The GOLD 5.0 molecular docking program was used to dock 20 HIV and HCV drugs against the ZIKV protease. Based on docking scores, 5 drugs were found to bind to the ZIKV protease with high affinities. Subsequently, the AMBER ff14SB force field was employed to simulate these drug-bound complexes of ZIKV protease. The MM/PBSA free energy method was utilized to compute the binding free energies of these complexes. Consequently, the two best ZIKV protease inhibitors were repurposed against the proteases of DENV and WNV.

**Results and Discussion:**

It is found that out of the 5 drugs, Ritonavir and Paritaprevir bind to the NS2B-NS3 protease of the ZIKV strongly with the Gibbs binding free energies (∆G_bind_) of −17.44±3.18 kcal/mol and −14.25±3.11 kcal/mol respectively. Remarkably, Ritonavir binds to the ZIKV Protease about 12 kcal/mol more strongly compared to its binding to the HIV protease. It is further found that Paritaprevir binds to DENV and WNV proteases as strongly as it binds to the ZIKV protease. Hence it is proposed that Paritaprevir may act as a potent pan-antiviral against the Zika, West Nile, and Dengue viral diseases.

## 1 Introduction

The flavivirus infections caused by the Zika virus (ZIKV), Dengue virus (DENV), and West Nile virus (WNV) cause mild to serious pathological conditions, such as fever, joint pain, shock, internal bleeding, organ failure, nausea, breathlessness, brain tissue damage, neurodegenerative diseases, and deaths (Broutet et al., 2016; Carod-Artal, 2018; de Araújo et al., 2018; Hersh et al., 2017; Kong et al., 2018; Mlakar et al., 2016; Moghadam et al., 2016; Musso and Gubler, 2016; Nash et al., 2001; Paixao et al., 2022). Unfortunately, no efficient vaccine or drug is available to date to prevent or treat these diseases in humans (Poland et al., 2018). Therefore, it is imperative to identify potential drug-like candidates that can be used to treat these diseases.

The ZIKV, DENV, and WNV possess structurally and functionally identical proteins (Nitsche, 2019; Wahaab et al., 2021; El Sahili et al., 2019; Chang et al., 2017). For example, the NS2B-NS3 proteases of these viruses are about 65% similar in amino acid sequence. The 3-dimensional structures of these proteins are also identical (Nitsche, 2019; Wahaab et al., 2021; El Sahili et al., 2019; Chang et al., 2017). Further, the main function of the NS2B-NS3 protease across these viruses is to cleave the polyprotein chain of these viruses to produce three structural (sp) and seven non-structural proteins (nsp) (Kuno et al., 1998; Falgout et al., 1991; Phoo et al., 2016; Zhang et al., 2016; Li and Kang, 2021). Therefore, it is necessary to inhibit the protease activities of these viruses to control their spread and subsequent deadly effects. It is now established that the NS2B-NS3 protease of these viruses contains identical folds and subdomains that directly or indirectly facilitate substrate binding and catalysis (Nitsche, 2019; Wahaab et al., 2021; El Sahili et al., 2019; Chang et al., 2017; Kuno et al., 1998; Falgout et al., 1991; Phoo et al., 2016; Zhang et al., 2016; Li and Kang, 2021). The substrate binding site consists of various sub-sites, namely, S1′, S2′, S3′, S1, S2, S3, and S4 (Zhang et al., 2016; Li and Kang, 2021; Robin et al., 2009; Li et al., 2018; Li et al., 2017; Pant and Jena, 2022). The primed sites facilitate catalysis, whereas the non-primed sites help accurately place the substrates into the active site and hold the substrates firmly till the completion of the catalysis (Pant and Jena, 2022). Therefore, identifying suitable inhibitors that can block the substrate binding sites of ZIKV, DENV, and WNV proteases will help inhibit the protease activities of these viruses. These inhibitors may further be developed as drug candidates for treating these viral diseases.

Although several attempts were made to identify potent inhibitors against these viral proteases individually (Zhang et al., 2016; Li and Kang, 2021; Robin et al., 2009; Li et al., 2018; Li et al., 2017; Pant and Jena, 2022; Phoo et al., 2018; Knox et al., 2006; Roy et al., 2017; Schüller et al., 2011; Behnam et al., 2015; Li et al., 2015; Grazia Martina et al., 2021; Song et al., 2021; Pushpakom et al., 2019; Pant et al., 2023; Pant and Jena, 2023), not a single drug has cleared the clinical trial. As the drug designing process is financially expensive and takes several years to discover a potent drug, drug repurposing is an efficient, less expensive, and prompt way of identifying existing drugs against these viral diseases (Talevi and Bellera, 2020; V Kleandrova and Speck-Planche, 2021; Cregar-Hernandez et al., 2011). Further, identifying common drugs against ZIKV, DENV, and WNV will substantially reduce the financial burden of designing new drugs and expedite the drug discovery process (Talevi and Bellera, 2020; V Kleandrova and Speck-Planche, 2021). For these reasons, the repurposing of 20 antiviral drugs (Table 1) recently proposed against proteases of the human immunodeficiency virus (HIV) (Wang et al., 2011; Tie et al., 2007; Walmsley et al., 2002; Klibanov et al., 2015; Koudriakova et al., 1998; Kempf and Sham, 1996) and hepatitis C virus (HCV) (Keating, 2016; Lam and Salazar, 2016; Shen et al., 2016; Kiang et al., 2013) is studied herein to evaluate their ability to bind to the substrate binding site of the NS2B-NS3 protease of the ZIKV. The two best drugs that have stable interactions with the ZIKV protease are repurposed against the WNV and DENV proteases (Yuan et al., 2017). Some of these drugs were also repurposed against ZIKV and SARS-COV-2 (Bolcato et al., 2020; Gurung et al., 2021; Jena, 2021). As these drugs were originally shown to inhibit the protease activities of HIV (Wang et al., 2011; Tie et al., 2007; Walmsley et al., 2002; Klibanov et al., 2015; Koudriakova et al., 1998; Kempf and Sham, 1996) and HCV (Keating, 2016; Lam and Salazar, 2016; Shen et al., 2016; Kiang et al., 2013), it is believed that these drugs may also inhibit proteases of other viral diseases due to their structural and functional similarities. For example, the catalytic residues of HCV (S139, H57, D81) are conserved in ZIKV (S135, H51, D75), WNV (S135, H51, D75), and DENV (S135, H51, D75) (Li and Kang, 2021; Robin et al., 2009; Li et al., 2018; Li et al., 2017; Pant and Jena, 2022; Phoo et al., 2018; Knox et al., 2006; Roy et al., 2017; Schüller et al., 2011; Behnam et al., 2015) and all of these proteases cleave the polypeptide chain to generate non-structural and structural proteins (Falgout et al., 1991; Phoo et al., 2016).

**TABLE 1:** Docking scores of different antiviral drugs bound to NS2B-NS3 of the ZIKV.

Sl. No	Ligands	ChemPLP score
1	Amprenavir	53.00
2	Saquinavir	69.67
3	Indinavir	61.23
4	Bevirimat	35.94
5	Nelfinavir	54.92
6	Fosamprenavir	55.76
7	Atazanavir	53.46
8	Lopinavir	59.43
9	Ritonavir	63.86
10	Darunavir	49.61
11	Danoprevir	40.42
12	Paritaprevir	58.53
13	Boceprevir	42.89
14	Simeprevir	51.73
15	Grazoprevir	43.57
16	Telaprevir	50.48
17	Dasabuvir	48.23
18	Asunaprevir	25.61
19	Glecaprevir	36.67
20	HZ-1157	42.17

To examine if these drugs can inhibit the ZIKV, WNV, and DENV proteases more effectively than their parent viral proteins (say, Ritonavir for HIV protease), the stability of Ritonavir bound to the HIV protease (PDB ID 1HXW) (Kempf et al., 1995) were compared with different complexes where Ritonavir is bound to ZIKV, WNV, and DENV proteases. Ritonavir was chosen because it effectively inhibits the HIV protease with good oral bioavailability in humans (Kempf et al., 1995).

## 2 Computational methodology

### 2.1 System preparation

**FIGURE 1:**
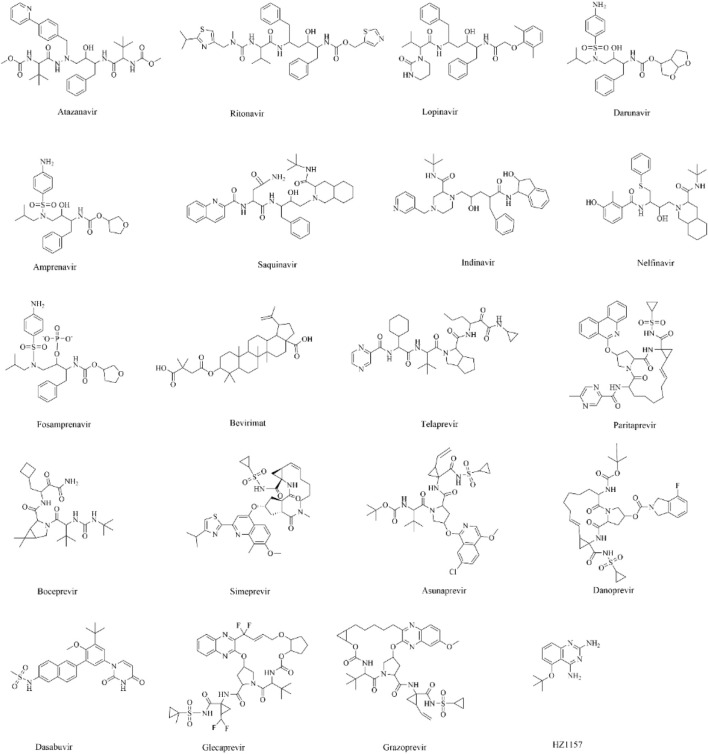
The two-dimensional structures of different drugs considered herein for molecular docking.

Initially, the high-resolution (2.0 Å) crystal structure of the NS2B-NS3 protease of the ZIKV bound to benzimidazol-1-yl-methanol (7HQ) (PDB ID 5H4I) (Zhang et al., 2016) was downloaded from the protein databank (https://www.rcsb.org/). Later, 7HQ was removed from the complex structure (PDB ID 5H4I) (Zhang et al., 2016) to generate the isolated protease structure. Subsequently, the missing ends of the protease were capped by NME (C-terminal) and ACE (N-terminal) molecules, and hydrogen atoms were added to the protease by using the GOLD 5.0 program (Jones et al., 1997; Li et al., 2014; Verdonk et al., 2003; Liebeschuetz et al., 2012). Consequently, 20 HIV and HCV drugs as summarized in Table 1 and Figure 1 were docked into the active site of the ZIKV protease by using the GOLD 5.0 program (Jones et al., 1997; Li et al., 2014; Verdonk et al., 2003; Liebeschuetz et al., 2012). The 3-dimensional structures of these drugs were downloaded from the DrugBank database (Wishart et al., 2018) and were energy-minimized before docking.

### 2.2 Molecular docking

To ensure that the docking program can accurately generate the protease-drug complex structures, the 7HQ was docked into the active site of NS2B-NS3 protease of the ZIKV to reproduce the experimental structure of the NS2B-NS3-7HQ complex (PDB ID 5H4I) (Zhang et al., 2016). The following docking protocols were used for this purpose. (1) The binding site was considered to be situated within a radius of 10 Å from the conserved Tyr161. (2) The genetic algorithm was used to create 10 different poses of each drug by using the ChemScore of GOLD 5.0 program (Jones et al., 1997; Li et al., 2014; Verdonk et al., 2003; Liebeschuetz et al., 2012). 100,000 genetic operations were performed to generate accurate poses. (3) The ChemPLP function was used to rank these 10 conformations based on different energy terms. (4) Although the drug molecules could adopt different conformations during docking, the protease was held rigid. The torsion angles of amino acids containing hydroxyl groups (Ser, Thr, and Tyr) were allowed to rotate to optimize their hydrogen bonding interactions. For the same reason, the Lysine NH_3_^+^ groups were allowed to rotate during docking. The ligand conformations were varied by not altering their bond lengths and angles. The stereochemistry of ligands was also not changed during docking. The atomic charges (formal and partial) were ignored during docking. Whether an atom is charged was decided by counting the bond orders and comparing the results with the atom’s normal valency (Jones et al., 1997; Li et al., 2014; Verdonk et al., 2003; Liebeschuetz et al., 2012). It should be mentioned that the ChemScore function was derived empirically from a set of 82 protein-ligand complexes for which measured binding affinities were available (Jones et al., 1997; Li et al., 2014; Verdonk et al., 2003; Liebeschuetz et al., 2012). However, the ChemPLP function uses Piecewise Linear Potential to model the steric complementarity between protein and ligand. Both functions calculate distance- and angle-dependent hydrogen bonding terms, lipophilic, and rotational energy terms. They also contain a clash penalty and internal torsion terms, which militate against close contacts in docking and poor internal conformations (Korb et al., 2009; Baxter et al., 1998) Similar docking protocols were used elsewhere (Saini et al., 2021; Saini et al., 2019).

Remarkably, the rank-1 pose of 7HQ was found to match its crystallographic conformation with a RMSD of 0.85 Å. The only difference between the docked and experimental complex structures arose from the adoption of different rotameric conformations of the C-OH bond of 7HQ (Pant and Jena, 2023). Therefore, the above docking protocols were used to produce different protease-drug complexes. Out of the 10 different conformations of each complex, the one that makes the maximum interactions with the protease and possesses a binding mode similar to that of 7HQ was short-listed for further analysis. This docking protocol was shown earlier to produce accurate docking poses (Pant and Jena, 2022; Pant et al., 2023; Pant and Jena, 2023; Pant and Jena, 2024).

It was found that among these 20 protease-drug complexes, 5 complexes possess the highest docking scores (Table 1). These complexes include drugs like Ritonavir, Saquinavir, Indinavir, Paritaprevir, and Lopinavir (Table 1). As the protease was held rigid and no solvent effects were considered during docking, it is expected that in the presence of water molecules and under the influence of protein dynamics, protease-drug complexes may undergo further conformational changes to make optimal interactions. Due to these reasons, the above 5 short-listed protein-drug complexes were subjected to molecular dynamics (MD) simulations. It should be mentioned that in an earlier *in vitro* structure-based drug discovery study (Yuan et al., 2017), the combination of Lopinavir-Ritonavir was found to inhibit the ZIKV replication by neutralizing the NS2B-NS3 protease. As the Lopinavir-Ritonavir combination is commercially available and commonly used clinically, the inhibition abilities of these individual drugs to the ZIKV protease were not evaluated (Yuan et al., 2017). Moreover, the binding mechanisms of these drugs to the ZIKV protease are also not known.

### 2.3 Molecular dynamics (MD) simulations

Before MD simulations, the short-listed docked complexes containing the ZIKV protease were solvated in a water box of size 10 Å. The TIP3P method (Mark and Nilsson, 2001; Onufriev and Izadi, 2018) was used to treat water molecules. Sufficient ions (Na^+^ and Cl^−^) were added to make the solvated complexes neutral. The GAFF method and AM1-BCC charge model were used to generate force fields for the drug molecules (Huang and Roux, 2013; He et al., 2020). The AMBER ff14SB force field (Maier et al., 2015) as implemented in the AMBER 14 program (Maier et al., 2015) was used to model the protease. Subsequently, each solvated neutral complex was energy minimized by 500 steps by using the steepest descent algorithm (Meza, 2010) followed by 1,000 steps by using the conjugate gradient algorithm (Štich et al., 1989). The minimizations were performed in three steps. In the first step, the water molecules were minimized by restraining the protease-drug complexes with a force constant of 50 kcal mol^-1^ Å^−2^. In the second step, the drug and water molecules were energy minimized by restraining the protease by a force constant of 50 kcal mol^-1^ Å^−2^. In the last step, all molecules were energy minimized without any restraints. Subsequently, each system was heated slowly to reach a temperature (T) of 300 K throughout 20 ps in the NVT ensemble. During this process, a force constant of 20 kcal mol^-1^ Å^−2^ was used to restrain the protease and drug molecules (except for their hydrogen (H) atoms). The temperature was regulated by using the weak-coupling algorithm (Berendsen et al., 1984). Later, a 100 ps of MD simulation was performed to equilibrate the system in the NPT ensemble while applying a harmonic restraint of 5 kcal mol^-1^ Å^−2^ at T = 300 K and a pressure (P) of 1.0 atm. The constant pressure of 1.0 atm was maintained using the Barendsen barostat (Ryckaert et al., 1977). Subsequently, each complex was equilibrated for 1,000 ps without restraining any molecules. During these processes, H atoms were constrained by using the SHAKE algorithm (Kräutler et al., 2001). The time step of MD simulations was set to 2 fs. A cutoff of 10 Å was set for non-bonded intermolecular interactions and long-range electrostatic interactions were treated by using the particle-mesh Ewald method (Darden et al., 1993). Consequently, each complex was subjected to a production run for 100 ns by using the NPT ensemble (T = 300 K and P = 1 atm).

As discussed later, among these drugs, Ritonavir and Paritaprevir made the most stable complexes with the ZIKA virus protease (Table 2). Therefore, to understand if these drugs can also bind strongly to the DENV and WNV proteases, the average simulated structures of the ZIKV protease-Ritonavir and ZIKV protease-Paritaprevir complexes were superimposed onto the crystal structures of WNV (PDB ID 2FP7) (Erbel et al., 2006) and DENV (PDB ID 3U1I) (Noble et al., 2012) proteases after removing crystallographic ligands bound to them. It should be mentioned that the amino acid sequences of PDB ID 3UI1 correspond to the DENV serotype 3 (DENV3) (Noble et al., 2012). Subsequently, the coordinates of Ritonavir and Paritaprevir were saved to generate the three-dimensional structures of the WNV protease-Ritonavir, WNV protease-Paritaprevir, DENV protease-Ritonavir, and DENV-Paritaprevir complexes. As the proteases of ZIKV, DENV, and WNV are structurally similar, it is believed that these drugs may bind to them by adopting a similar mechanism. However, the protease dynamics may induce conformational changes in the drugs, which can be captured by using the MD simulation techniques. Therefore, the generated protease-drug complexes were initially energy minimized, heated to 300 K, equilibrated for 1000 ps, and subjected to production run for 100 ns by employing all the steps mentioned above for the ZIKV.

**TABLE 2:** The average root mean square deviations (RMSD) of the Cα atoms and the average radius of gyration (Rg) of the NS2B-NS3 protease in ligand-free and ligand-bound conformations.

S.No.	Complex	Average RMSD (Å)	Average Rg (Å)
1	ZIKV Protease-ligand free	1.394	16.45
4	ZIKV Protease-Ritonavir	1.547	16.40
5	ZIKV Protease-Saquinavir	1.419	16.51
6	ZIKV Protease-Indinavir	1.391	16.55
7	ZIKV Protease-Paritaprevir	1.442	16.40
8	ZIKV Protease-Lopinavir	1.437	16.32
9	WNV Protease-ligand free	1.592	16.03
10	WNV Protease-Ritonavir	1.402	16.01
11	WNV Protease-Paritaprevir	1.559	16.04
12	DENV Protease-ligand free	2.286	16.88
13	DENV Protease-Ritonavir	1.840	16.82
14	DENV Protease-Paritaprevir	2.162	16.89

To compare the structures of the NS2B-NS3-drug complexes with the ligand-free structures of the NS2B-NS3 proteases, the active conformations of ZIKV (PDB ID 5H4I) (Zhang et al., 2016), WNV (PDB ID 2FP7) (Erbel et al., 2006), and DENV (PDB ID 3UI1) (Noble et al., 2012) proteases were simulated for 100ns each after removing their crystallographic ligands from their binding sites. As in the apo (inactive) conformations of WNV (PDB ID 2GGV) (Aleshin et al., 2007) and DENV proteases (PDB ID 2FOM) (Erbel et al., 2006), the NS2B is protruding to the solvent and is placed away from the active site of the NS3 (Supplementary Figure S4), these structures cannot facilitate the substrate binding. Therefore, the comparison of MD-simulated ligand-bound conformations of the ZIKV, WNV, and DENV proteases with their apo structures would not yield meaningful results. For this reason, the ligand-free structures of these viral proteases were simulated to compare with the simulated drug-bound conformations of different viral proteases. The HIV protease-Ritonavir complex (PDB ID 1HXW) (Kempf et al., 1995) was also simulated for 100ns to compare its binding free-energy data with the ZIKV-Ritonavir, WNV-Ritonavir, and DENV-Ritonavir complexes.

### 2.4 Binding free energy calculations

The Gibb’s binding free energy $\left(∆G_{\text{bind}}\right)$ for each protease-drug complex was calculated by using the molecular mechanics energies combined with the Poisson–Boltzmann surface area (MM/PBSA) continuum solvation methods (Homeyer and Gohlke, 2012) as implemented in the AMBER 14 package (Case et al., 2014). For this purpose, 100 snapshots were extracted from the last 10 ns MD trajectories at an interval of 100 ps by stripping water molecules and ions. The binding free energy was calculated by using Equation 1 (Hou et al., 2011; Wang et al., 2019).

$$∆G_{\mathrm{bind}}=G_{\mathrm{complex}\;\left(\mathrm{minimized}\right)}‐G_{\mathrm{protein}\;\left(\mathrm{unbound},\mathrm{minimized}\right)}‐G_{\mathrm{ligand}\;\left(\mathrm{unbound},\mathrm{minimized}\right)}$$ (1)

where $∆G_{\text{bind}}$ is the calculated binding free energy, $G_{\text{complex}\;\left(\text{minimized}\right)}$ is the MM/PBSA energy of the minimized complex, $G_{\text{protein}\;\left(\text{unbound},\text{minimized}\right)}$ is the MM/PBSA energy of the protein after separating it from its bound ligand, and $G_{\text{ligand}\;\left(\text{unbound},\text{minimized}\right)}$ is the MM/PBSA energy of the ligand after separating it from complex and allowing it to relax. Due to computational complexity, we have not considered normal mode analysis. Therefore, the $∆G_{\text{bind}}$ values do not contain entropy contributions.

## 3 Results and discussion

**FIGURE 2:**
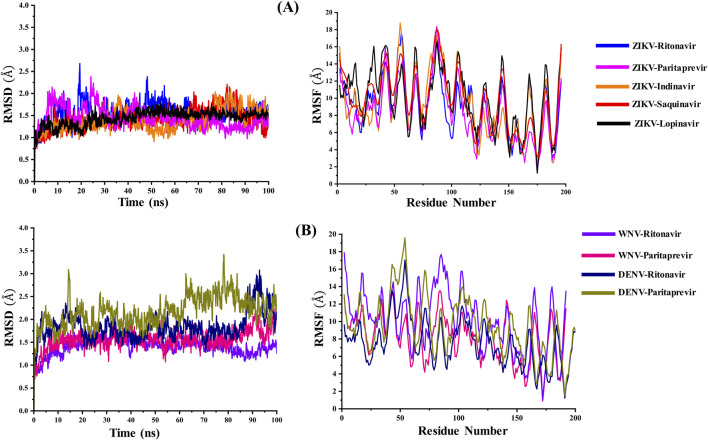
**(A)** The variations in the root mean square deviations (RMSD in Å) of the Cα atoms and the root mean square fluctuations (RMSF in Å) of amino acid residues of the ZIKV NS2B-NS3 protease bound to different ligands with simulation time. **(B)** The variations in the RMSD (Å) of the Cα atoms and the RMSF (Å) of amino acid residues of the WNV and DENV NS2B-NS3 proteases bound to Ritonavir and Paritaprevir with simulation time. These values were calculated by considering the minimized structure as the reference.

**FIGURE 3:**
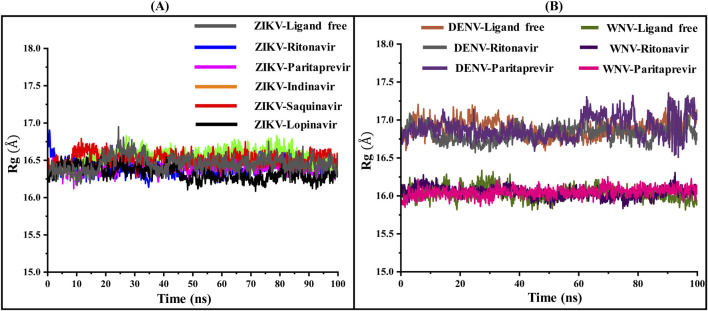
The variations in the radius of gyration (Rg in Å) of the **(A)** ZIKV and **(B)** WNV, and DENV NS2B-NS3 proteases in ligand-free and ligand-bound conformations with simulation time.

The root mean square deviations (RMSD in Å) of C_α_ atoms of the ZIKV, WNV, and DENV proteases and the root mean square fluctuations (RMSF in Å) of different residues of these proteases are illustrated in Figure 2. Their radius of gyration are illustrated in Figure 3. The average RMSD of the of C_α_ atoms and the average radius of gyration of these virals proteases are presented in Table 2. From Figures 2, 3 and Table 2, it is clear that the average RMSD is less than 2.0 Å for all proteases studied herein and hence the ligand binding to them would stabilize their structure. Although RMSD of ZIKV protease-Lopinavir complex slightly increased from the average value between 60–80 ns, it decreased afterward. It could be due to conformational changes occurring in the ZIKV-protease due to Lopinavir binding. The computed radius of gyration of all complexes suggests that the binding of different drugs to the active site of the viral proteases would help the protease to acquire a compact structure (Figure 3). It should be mentioned that the ligand-free structures of the viral proteases possess the similar Rg values as computed for the protease-drug complexes (Table 2; Figure 3). This is because the ligand-free structures were derived from the ligand-bound complex structures after removing crystallographic ligands, where the protease had already acquired a compact structure (Zhang et al., 2016; Erbel et al., 2006; Noble et al., 2012).

**FIGURE 4:**
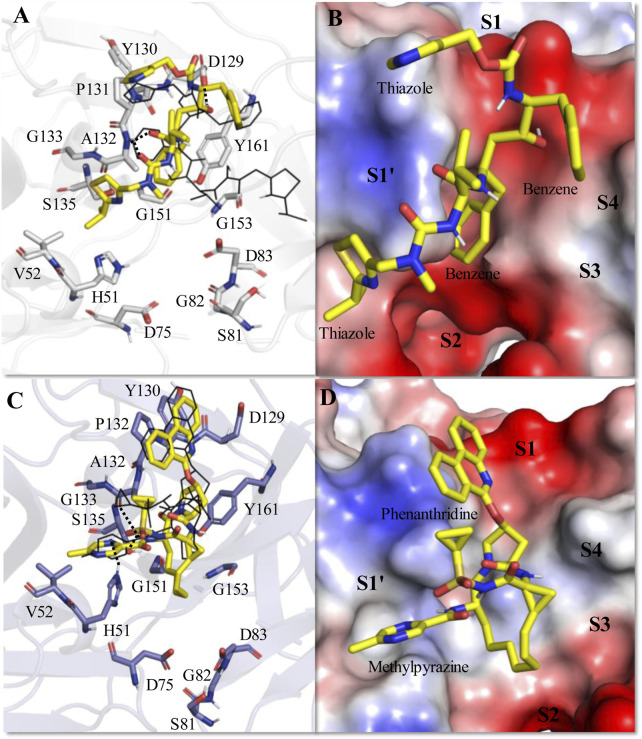
Average MD-simulated structures of **(A, B)** ZIKV NS2B-NS3-Ritonavir and **(C, D)** ZIKV NS2B-NS3-Paritaprevir complexes. The initial docking conformations of Ritonavir and Paritaprevir are shown in lines (in black color). These drugs bound to the electrostatic potential surface of the NS2B-NS3 protease and different substrate sites (S1–S4 and S1′) of the protease are also shown **(B, D)**. The red and blue colors refer to the negative and positive electrostatic potential regions respectively. Important rings of Ritonavir and Paritaprevir are mentioned. Hydrogen bonding interactions are shown by dotted lines.

**FIGURE 5:**
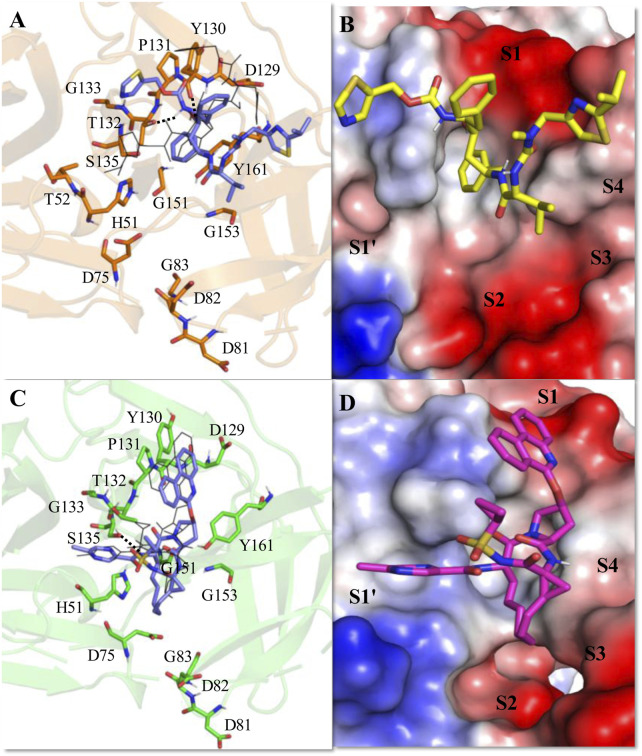
Average MD-simulated structures of **(A, B)** WNV NS2B-NS3-Ritonavir and **(C, D)** WNV NS2B-NS3-Paritaprevir complexes. The initial conformations of Ritonavir and Paritaprevir (generated after superimpositions of ZIKV protease-Ritonavir and ZIKV protease-Paritaprevir complexes onto the crystal structure of WNV protease (PDB ID (2FP7)) are shown in lines (in black color). Hydrogen bonding interactions are shown by dotted lines. These drugs bound to the electrostatic potential surface of the NS2B-NS3 protease and different substrate sites (S1–S4 and S1′) of the protease are also shown **(B, D)**. The red and blue colors refer to the negative and positive electrostatic potential regions respectively.

**FIGURE 6:**
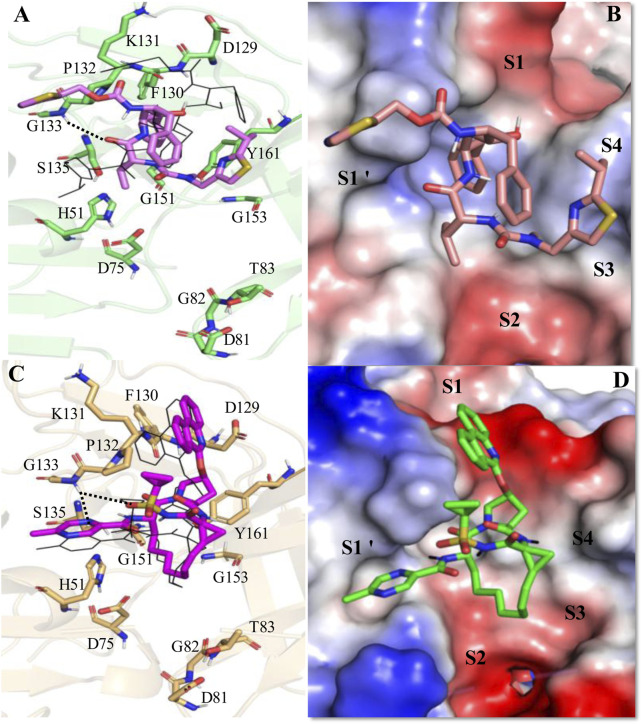
Average MD-simulated structures of **(A, B)** DENV NS2B-NS3-Ritonavir and **(C, D)** DENV NS2B-NS3-Paritaprevir complexes. The initial conformations of Ritonavir and Paritaprevir (generated after superimposition of the ZIKV protease-Ritonavir and ZIKV protease-Paritaprevir complexes onto the crystal structure of DENV protease (PDB ID 3U1I)) are shown in lines (in black color). Dotted lines show hydrogen bonding interactions. These drugs bound to the electrostatic potential surface of the NS2B-NS3 protease and different substrate sites (S1–S4 and S1′) of the protease are also shown **(B, D)**. The red and blue colors refer to the negative and positive electrostatic potential regions respectively.

The average MD-simulated structures of different complexes are shown in Figures 4–6 and Supplementary Figures S1–S3. Important interactions made between the drug molecules and different residues of these proteases are also depicted in these Figures (Figures 4–6; Supplementary Figures S1–S3). From these figures, it is evident that the short-listed drug molecules are well placed in the active site of the protease and are making stable interactions with them. The stability of these complexes is also evident from the negative ΔG_bind_ values (Table 3; Supplementary Table S1). It should be mentioned that the hydrogen bonding interactions that lasted for >80% (occupancy >80%), 50%–79% (occupancy 50%–79%), and <50% of the simulation time (occupancy <50%) are considered to be strong, moderate, and weak respectively (Pant and Jena, 2022; Pant et al., 2023). The π-π stacking interaction between two aromatic rings is considered to be strong if the distance between them is <3.5 Å (Riwar et al., 2017).

**TABLE 3:** Gibb’s binding free energies (ΔG_bind_) of different protease-drug complexes obtained by using the MM/PBSA method.

Sl. No.	Complexes	ΔG_bind_ (kcal/mol)
1	ZIKV Protease-Ritonavir	−17.44 ± 3.18
2	ZIKV Protease-Paritaprevir	−14.25 ± 3.11
3	ZIKV Protease-Lopinavir	−7.34 ± 2.56
4	ZIKV Protease-Saquinavir	−5.03 ± 2.98
5	ZIKV Protease-Indinavir	−2.95 ± 2.30
6	WNV Protease-Ritonavir	−7.43 ± 2.16
7	WNV Protease-Paritaprevir	−17.3 ± 2.55
8	DENV Protease-Ritonavir	−11.51 ± 2.82
9	DENV Protease-Paritaprevir	−12.76 ± 2.91
10	HIV Protease-Ritonavir	−5.84 ± 3.93

### 3.1 Drug bindings to the ZIKV protease

If we compare the ΔG_bind_ values of different drugs bound to the ZIKV protease, they follow the order Ritonavir > Paritaprevir > Lopinavir > Saquinavir > Indinavir (Table 3). The contributions of different energies to ΔG_bind_ indicate that the higher stabilities of Ritonavir and Paritaprevir arise mainly due to the favorable van der Wall and non-polar interactions with the protease (Supplementary Table S1). Therefore, Ritonavir and Paritaprevir may act as potent inhibitors of the NS2B-NS3 protease of the ZIKV.

The average MD simulated structure of the ZIKV protease-Ritonavir complex indicates that the tail thiazole ring of Ritonavir is quite mobile and adopts a different conformation than its original docked conformation (Figures 4A, B). During the MD simulation, the tail thiazole ring of Ritonavir moved from the S3 site and was placed in between the S2 and S1′ sites. The head thiazole ring of Ritonavir is also slightly moved away from the S1 pocket (Figure 4B). In this conformation, the head and tail thiazole rings of Ritonavir make a T-shaped stacking interaction with P131 and V52 respectively. It also makes two weak hydrogen bonds (<50% occupancy) with D129, and A132 of the protease (Figure 4A). One of its benzene rings makes a stable π-π stacking interaction with Y161 and the tail thiazole ring makes hydrophobic interactions with A132 and G133. This indicates that Ritonavir is tightly bound to the protease, which is evident from its ΔG_bind_ of −17.44 ± 3.18 kcal/mol (Table 3). This complex is about 12 kcal/mol more stable than the HIV-Ritonavir complex (Table 3). This suggests that Ritonavir would bind to the ZIKV protease more strongly than that of HIV protease, and therefore, it may act as a better drug to inhibit the ZIKV protease activities. Interestingly, the docked structure of Paritaprevir did not change much in the MD simulation. Its phenanthridine ring is placed in the S1 site and the methylpyrazine ring is placed in the S1′ site as was obtained in the docked conformation (Figures 4C, D). In this conformation, it makes two strong π-π interactions with P132, and Y161 and several hydrophobic interactions with different residues of the protease (Figure 4C). Its tail methyl pyrazine ring can make a transient hydrogen bond with H51 and the NH group can make two hydrogen bonds with S135 and A132 (Figure 4C). For these reasons, its ΔG_bind_ is −14.25 ± 3.11 kcal/mol, which is only about 3 kcal/mol less stable than that of the ZIKV protease-Ritonavir complex (Table 3).

During MD-simulation, Lopinavir slightly moved away from its initial docking conformation mainly because of the rotation of its dimethylphenoxy group away from the S1 site (Supplementary Figure S1). However, its tetrahydropyrimidine ring remained intact in its initial position. Because of this, the latter group made one moderate hydrogen bond with Y161 (62% occupancy), and two weak hydrogen bonds with G151 (34% occupation), and G153 (30% occupation). Because of these interactions, its ΔG_bind_ is calculated to be −7.34 ± 2.56 kcal/mol, which is about 10 kcal/mol less negative than that of the ZIKV protease-Ritonavir complex (Table 3). The MD-simulated structure of Saquinavir is similar to its docking structure (Supplementary Figure S2). Although it remains within the binding pocket of the protease, it does not make any stable hydrogen bond with the protease (Supplementary Figure S2). Its stability is mainly governed by two weak π-π interactions with 161 of the protease. For this reason, its ΔG_bind_ is computed to be −5.03 ± 2.98 kcal/mol, which is about 12 kcal/mol less stable than that of the ZIKV-protease-Ritonavir complex (Table 3). Interestingly, during MD simulation, Indinavir adopted a folded confirmation (Supplementary Figure S3), wherein it makes two weak hydrogen bonds (<50% occupancy) with S135 and H51. However, it failed to make the π-π stacking interaction with Y161 (Supplementary Figure S3). It is also unable to make any hydrophobic interactions with the protease. For this reason, its binding free energy is the lowest (−2.95 ± 2.30 kcal/mol) among all drugs studied herein (Table 3).

It should be mentioned that in an earlier experimental study (Li et al., 2018), a small molecule ligand (5-amino-1-((4-methoxyphenyl)sulphonyl)-1H-pyrazol-3-yl benzoate) was found to covalently bound to the ZIKV protease. Although the complete structure of the ligand could not be crystallized, the X-ray study (Li et al., 2018) resolved a fragment of the ligand (benzoyl moiety), occupying the S1 site of the protease. In this conformation, the benzoyl moiety was found to make a π-π -stacking interaction with Y161 in similar manner as obtained for Paritaprevir and other ligands. Another small molecule ligand (1H-benzo[d]imidazole-1-yl methanol) was also observed to be stabilized by making a π-π -stacking interaction with Y161 (Zhang et al., 2016)**.**

### 3.2 Bindings of Ritonavir and Paritaprevir to the WNV protease

In the active site of the WNV protease, Ritonavir adopted a new conformation (Figures 5A, B), which is significantly different from its conformation adopted in the active site of ZIKV protease (Figures 4A, B). This happened because of the rotation of the head thiazole group of Ritonavir away from the S1 site toward the S1′ site (Figures 5 A, B). Similarly, the tail thiazole ring moved toward the S4 site. Because of these, it failed to interact with P131 and T52 of WNV protease as noticed in the case of ZIKV protease (Figure 4A). This conformation is mainly stabilized by a strong π-π stacking interaction with Tyr161 and transient hydrogen bonds with T132 and Y130 (Figure 5A). For these reasons, a ΔG_bind_ of −7.43 ± 2.16 kcal/mol is obtained for the WNV-protease-Ritonavir complex (Table 3). Therefore, Ritonavir is loosely bound to the WNV protease compared to that of ZIKV protease. However, its binding to WNV protease is about 2 kcal/mol more stable than its binding of HIV protease (Table 3). Interestingly, Paritaprevir is bound to the WNV protease in a similar manner as it binds to the ZIKV protease (Figures 5 C, D). Its binding to the WNV protease is mainly stabilized by strong π-π stacking interactions with Tyr161 and Pro131 (Figures 5 C, D). Its NH group makes a transient hydrogen bond with S135. It also makes several hydrophobic interactions with the protease. Its phenanthridine ring is placed in the S1 site and the methyl pyrazine ring is bound to the S1′ site. As the catalytic reactions are induced by the residues of the S1′ site, the placement of the methyl pyrazine ring of Paritaprevir in this site would affect the protease activities severely. Interestingly, a ΔG_bind_ of −17.3 ± 0.25 kcal/mol is obtained for the WNV-Paritaprevir complex (Table 3), which is about 3 kcal/mol more stable than that of the ZIKV-Paritaprevir complex. This suggests that Paritaprevir binds to the WNV protease more strongly compared to the ZIKV protease.

### 3.3 Bindings of Ritonavir and Paritaprevir to DENV protease

In the substrate binding site of DENV protease (Figure 6), Ritonavir undergoes a similar conformational change as noticed in the case of WNV protease (Figures 5A, B). However, it adopted a somewhat compact and folded structure compared to the extended conformation obtained in the case of ZIKV (Figure 4A) and WNV proteases (Figure 5A). It is mainly stabilized by making a weak hydrogen bond with G133 and π-π stacking interactions with P132, Y161, and V155 (Figures 6A, B). For these reasons, a ΔG_bind_ of −11.51 ± 2.82 kcal/mol is obtained for the DENV-Ritonavir complex, which is about 3 kcal/mol more stable than that of the WNV protease-Ritonavir complex and about 6 kcal/mol more stable than the HIV-Ritonavir complex (Table 3). This indicates that Ritonavir would act as a better inhibitor of DENV protease compared to WNV and HIV proteases. However, as the ZIKV protease-Ritonavir complex is about 6 kcal/mol more stable than that of the Ritonavir-DENV protease complex (Table 3), Ritonavir would be more potent against the ZIKV protease. Interestingly, the binding mode of Paritaprevir (Figures 6C, D) is identical to its binding mode obtained in the case of ZIKV and WNV proteases. As Paritaprevir contains more hydrophobic groups, is heavier compared to Ritonavir, and contains a cyclic ring, it does not move much during the MD simulations. Further, due to strong π-π stacking interactions, it remains intact within the protease binding site. It is found that in the DENV protease active site, the head phenanthridine ring of Paritaprevir maintains its π-π stacking interaction with Y161 (Figures 6C, D). However, as DENV protease contains K131, (instead of P131 of ZIKV) its Phenanthridine ring failed to make another strong π-π stacking interaction with K131. Despite this, it made a relatively weak stacking interaction with P132 (Figure 6C). The tail methyl pyrazine ring and the NH group of Paritaprevir can also make weak hydrogen bonds with G133 (Figure 6C). As a result, a ΔG_bind_ of −12.76 ± 2.91 kcal/mol is obtained for this complex, which is about 1 kcal/mol less stable than the ZIKV protease-Paritaprevir complex and about 4 kcal/mol less stable than that of the WNV protease-Paritaprevir complex (Table 3). These results indicate that Paritaprevir binds most strongly to the WNV protease and moderately strongly to the ZIKV and DENV proteases.

### 3.4 Comparison of structures and dynamics of ZIKV, WNV, and DENV proteases bound to Ritonavir and Paritaprevir

**FIGURE 7:**
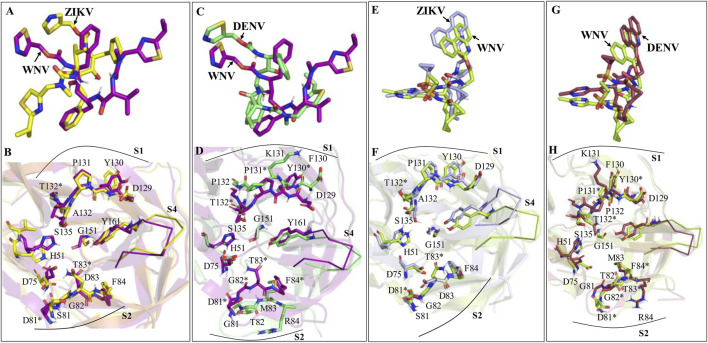
**(A, B)** Superimpositions of the WNV-Ritonavir complex onto the ZIKV-Ritonavir complex. **(C, D)** Superimpositions of the DENV-Ritonavir complex onto the WNV-Ritonavir complex. **(E, F)** Superimpositions of the WNV-Paritaprevir complex onto the ZIKV-Paritaprevir complex. **(G, H)** Superimpositions of the DENV-Paritaprevir complex onto the WNV-Paritaprevir complex. Some of the residues of ZIKV, WNV, and DENV are common. However, distinct residues of WNV (not present in ZIKV and DENV) are marked by *. Residues of S1 and S2 sites are shown by sticks and residues of S4 sites are shown by ribbons.

If we compare the structures of Ritonavir bound to different proteases, it appears that Ritonavir is quite flexible and can adopt different conformations within the active site of ZIKV, WNV, and DENV proteases (Figure 7). However, its binding to the ZIKV protease is stronger compared to other viral proteases considered here. Although the S1 site residues of ZIKV (D129, Y130, P131, A132) and WNV (D129, Y130, P131, T132) possess identical sequences and structures (Figures 7A, B), the less binding affinity of Ritonavir toward the WNV protease is presumably because of the inward movements of its S2 (S81-G83) and S4 site residues (V153-Y161) that push Ritonavir to move away from the active site (Figures 7 A, B). However, in the case of DENV, the S2 and S4 site residues moved away from the active site, thereby, providing Ritonavir more space to undergo further conformational changes (folding conformation). Further, as the S1 site (D129, F130, K131, P132) and S2 site residues (D81, G82, T83, and M84) of the DENV are significantly different from those of WNV in sequence and structure (Figures 7 C, D), Ritonavir faces different protein dynamics in the active site of these proteases. This ultimately produces different stabilities for Ritonavir (Table 3). However, interestingly, except for the S4 site residues, other binding site residues of ZIKV and WNV proteases do not move much during the binding of Paritaprevir to them (Figures 7 E, F). This is also true for the DENV protease (Figures 7G, H). We also noted that the binding of Paritaprevir to WNV and DENV proteases stabilizes the S2 site residues (Figures 7G, H) unlike in the case of the binding of Ritonavir (Figures 7C, D).

**FIGURE 8:**
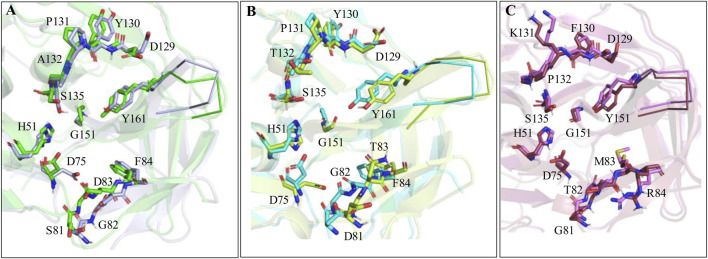
**(A)** Superimposition of ZIKV protease-Paritaprevir complex structure onto the crystal structure of ZIKV protease (PDB ID 5H4I). **(B)** Superimposition of WNV-protease-Paritaprevir complex onto the crystal structure of the WNV protease (PDB ID 2PF7). **(C)** Superimposition of DENV-Paritaprevir complex onto the crystal structure of DENV protease (PDB ID 3U1I).

To further understand the role of protein dynamics in the ligand binding to the proteases of ZIKV, WNV, and DENV, the simulated complex structures of Paritaprevir bound to these proteases were superimposed to the crystal structures of ZIKV-7HQ (PDB ID 5H4I) (Zhang et al., 2016), WNV-benzoyl-Nle-K-R-R-H (PDB ID 2FP7) (Erbel et al., 2006) and DENV-tripeptide (PDB ID 3U1I) (Noble et al., 2012) complexes (Figures 8A–C). It is found that mainly S2 and S4 site residues of the ZIKV and WNV proteases undergo conformational changes during the ligand binding (Figures 8A, B) and a minor conformational change occurs in the S4 site residues of the DENV protease (Figure 8C). Moreover, important catalytic residues, such as S135, H51, and D75 are found to adopt different conformations depending on the nature of the ligand entering into the active site of the ZIKV and WNV proteases. These results indicate that active site residues of DENV are more rigid compared to those of ZIKV and WNV, and therefore, DENV would respond equally to different ligands irrespective of their nature (small molecules or peptides or peptidomimetics) (Zhang et al., 2016; Li and Kang, 2021; Robin et al., 2009; Li et al., 2018; Li et al., 2017; Pant and Jena, 2022; Phoo et al., 2018; Knox et al., 2006; Roy et al., 2017; Schüller et al., 2011; Behnam et al., 2015; Li et al., 2015; Grazia Martina et al., 2021; Song et al., 2021; Pushpakom et al., 2019; Pant et al., 2023; Pant and Jena, 2023).

We also noticed that Paritaprevir binds to the active site of ZIKV, WNV, and DENV proteases similarly as observed for the 7HQ and peptide ligands (Supplementary Figure S4). In an earlier study (Viswanathan et al., 2014) about 2211 different DENV strains from four different DENV sterotypes were compared. It was found that about 40% of residues of DENV are invariant, while about 66% of active site amino acid sequences are identical (Viswanathan et al., 2014). Therefore, Paritaprevir may bind to all strains and sterotypes of DENV similarly as obtained here for DENV3 protease. Therefore, it may inhibit the activities of all DENV stereotypes. It should be mentioned that as the substrate binding site of these proteases is acidic in nature, peptide or peptidomimetic inhibitors containing basic residues were thought to be effective against these proteases (Viswanathan et al., 2014). However, the highly negative ΔG_bind_ values obtained here (Table 2) suggest that neutral and small molecule ligands like Ritonavir and Paritaprevir may also inhibit the protease activities of ZIKV, WNV, and DENV effectively. Among the two, Paritaprevir may act as a pan-antiviral against the Zika, Dengue, and West Nile viral diseases due to its strong binding with their NS2B-NS3 proteases. Interestingly, as Paritaprevir in combination with other drugs has already been approved for its use against different hepatitis C virus genotypes (Klibanov et al., 2015; Keating, 2016; Hussaini, 2016; Menon et al., 2016), it would be safe for humans and can be repurposed against ZIKV, DENV, and WNV viral diseases. However, as the computational results obtained herein are force-field dependent and no wet-lab experiments were performed to validate the computational findings, it is necessary to verify the efficacy of Paritaprevir against ZIKV, DENV, and WNV proteases by conducting *in vitro* enzymatic assays or viral replication studies.

## 4 Conclusion

Among the five screened drugs, such as Ritonavir, Saquinavir, Indinavir, Paritaprevir, and Lopinavir, the anti-HIV drug Ritonavir (ΔG_bind_ = −17.44 ± 3.18) and the anti-HCV drug Paritaprevir (ΔG_bind_ = −14.25 ± 3.11) are found to bind to the ZIKV NS2B-NS3 protease quite strongly. Remarkably, Ritonavir is found to bind to ZIKV protease significantly more strongly than that of the HIV Protease. Therefore, it is proposed that the repurposing of these drugs against the ZIKV may potentially decrease the virus loads in patients. It is further found that Paritaprevir binds to WNV protease even more strongly than ZIKV protease with a ΔG_bind_ = −17.3 ± 2.55 kcal/mol. Hence, Paritaprevir may also act as a potent drug to reduce the virus loads in patients suffering from West Nile Viral disease. However, Ritonavir may not be a good drug candidate against the WNV protease due to its relatively loose binding to the WNV protease (ΔG_bind_ = −7.43 ± 2.16). Due to the moderately strong binding of Ritonavir (ΔG_bind_ = −11.51 ± 2.82) and Paritaprevir (ΔG_bind_ = −12.76 ± 2.91) to the DENV protease, they may be effective for patients suffering from Dengue viral disease. These results also indicate that Paritaprevir, due to its better binding with proteases of ZIKV, WNV, and DENV, may act as a pan-antiviral against these viral diseases. However, experimental biochemical studies evaluating the binding and toxicological effects of these drugs are necessary before confirming their use against these viral diseases. Nevertheless, this study is expected to aid in the further design and analysis of drugs against these viral diseases considering the main scaffolds of Ritonavir and Paritaprevir.

## Data Availability

The original contributions presented in the study are included in the article/Supplementary Material, further inquiries can be directed to the corresponding author.
