# Supplementary material for: Paritaprevir as a pan-antiviral against different flaviviruses
Source: Front Mol Biosci. 2025 Apr 3;12:1524951. doi: 10.3389/fmolb.2025.1524951 (PMC12003128; doi:10.3389/fmolb.2025.1524951)
Supplement: Supplementary file 1 [file Presentation1.pdf]

Supporting Information

Paritaprevir as a Pan-antiviral Against Different Flaviviruses

R.P. Yadav[a] and N.R. Jena[a]*

[a]Discipline of Natural Sciences, Indian Institute of Information Technology, Design and Manufacturing, Dumna Airport Road, Jabalpur-482005, India

Corresponding Author's Email Address: nrjena@iiitdmj.ac.in

Table S1: The MM/PBSA binding free energies (ΔG_{bind}) of different complexes and contributions of different energies to the ΔG_{bind}. All energies are provided in kcal/mol.

Sl No	Complexes	ΔG_{bind}	ΔE_{vdw}	ΔE_{Elec}	ΔE_{PB}	ΔE_{NP}	ΔE_{Disp}
1	ZIKV-Ritonavir	-17.4±3.18	-44.2±3.23	-7.43±2.89	11.52±2.8	-30.55±2.05	53.2±2.41
2	ZIKV-Saquinavir	-5.03±2.90	-34.7±3.23	-0.57±1.65	7.48±1.61	-22.11±1.93	46.8±2.52
3	ZIKV-Indinavir	-2.95±3.55	-15.4±5.9	-2.03±2.19	3.79±1.8	-12.06±4.86	22.82±7.40
4	ZIKV-Paritaprevir	-14.2±3.14	-48.8±2.84	-7.16±1.20	13.2±1.30	-29.94±1.36	58.45±1.96
5	ZIKV-Lopinavir	-7.33±2.56	-30.8±3.03	-7.3±1.26	9.72±1.34	-22.40±2.13	43.54±2.76
6	WNV-Ritonavir	-7.43±2.16	-30.64±1.92	-2.07±0.58	6.58±0.57	-22.0±1.15	40.79±1.29
7	WNV-Paritaprevir	-17.3±2.55	-51.5±2.75	-6.74±1.11	12.40±1.19	-33.4±1.39	61.9±2.05
8	DENV-Ritonavir	-11.5±2.82	-39.32±3.25	-5.24±1.28	9.08±1.40	-27.6±2.30	51.6±3.00
9	DENV-Paritaprevir	-12.7±2.91	-51.7±3.88	-4.29±1.04	11.7±1.53	-32.08±2.17	63.6±3.34

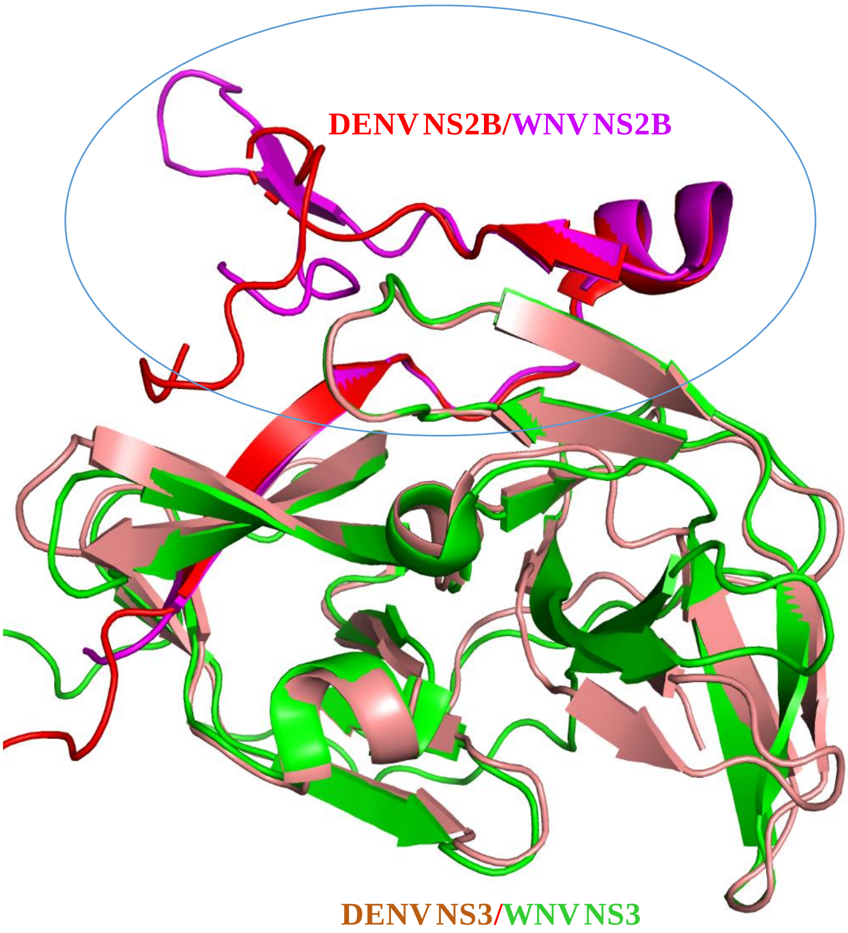

Fig. S1: Comparison of N2B (marked in a circle) and NS3 proteins of the WNV and DENV Proteases.

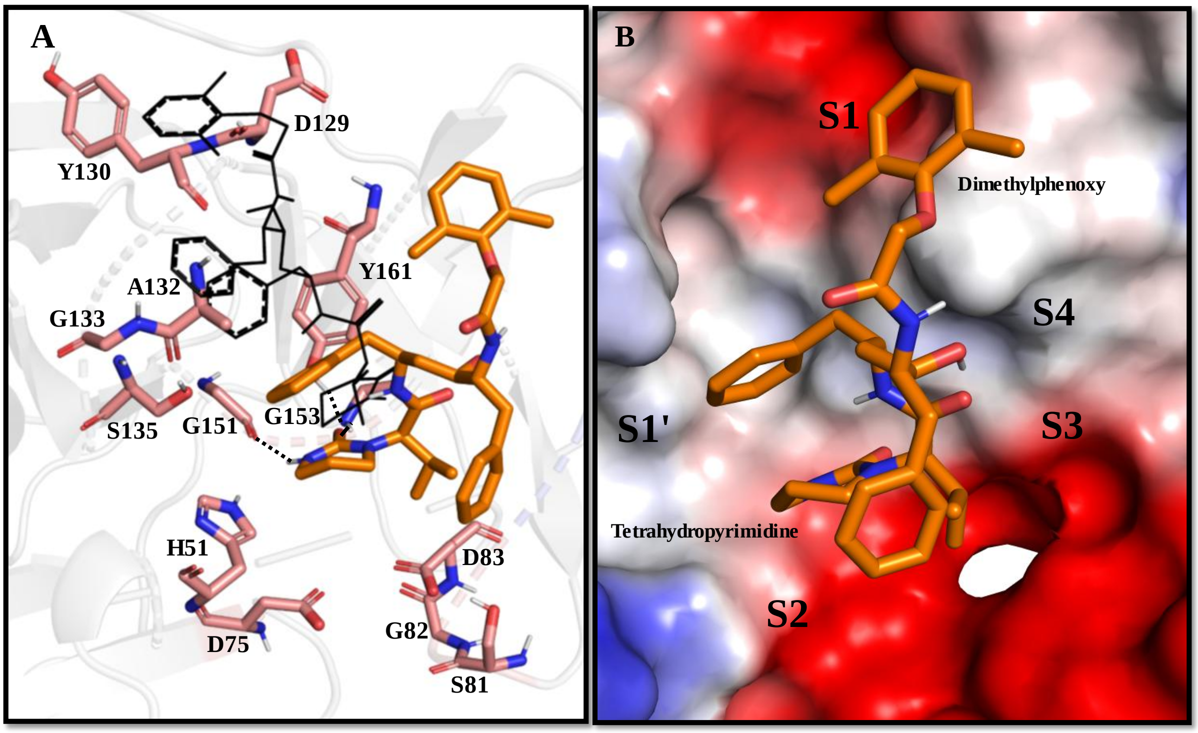

Fig. S2: Average MD-structure of ZIKV protease-Lopinavir complex. (A) Interaction of Lopinavir with different residues of the ZIKV protease and (B) Placement of Lopinavir in the electrostatic surface of the protease.

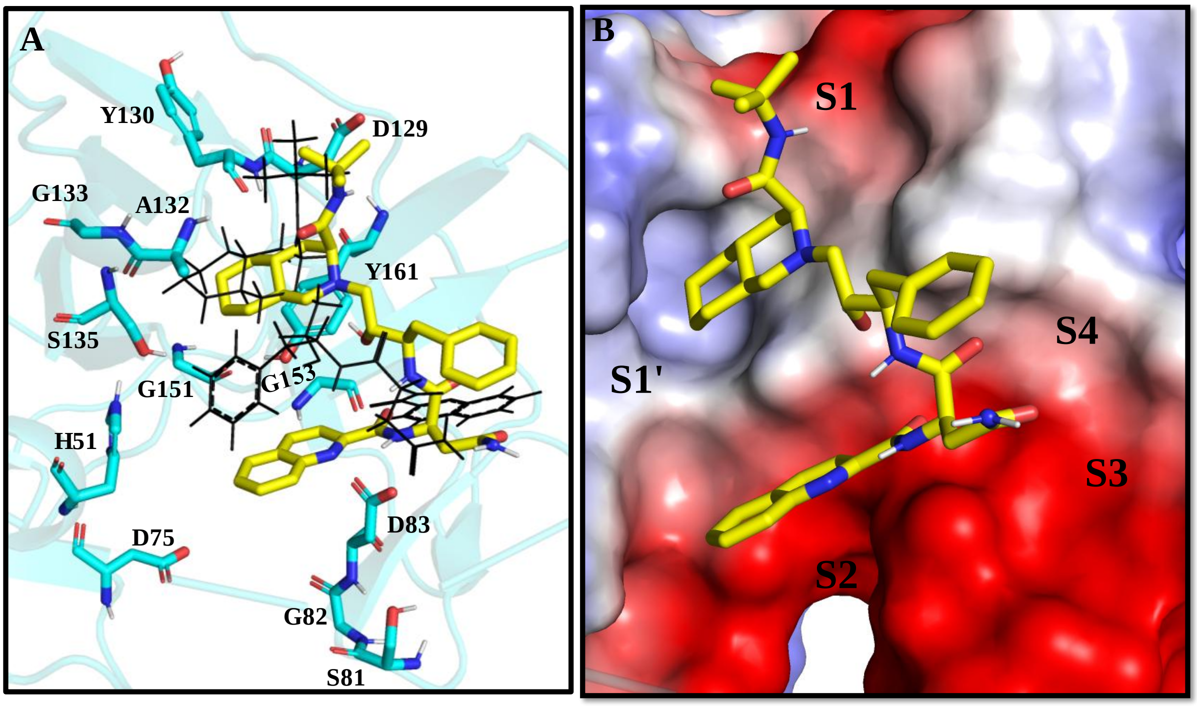

Fig. S3: Average MD-structure of ZIKV protease-Saquinavir complex. (A) Interaction of Saquinavir with different residues of the ZIKV protease and (B) Placement of Saquinavir in the electrostatic surface of the protease.

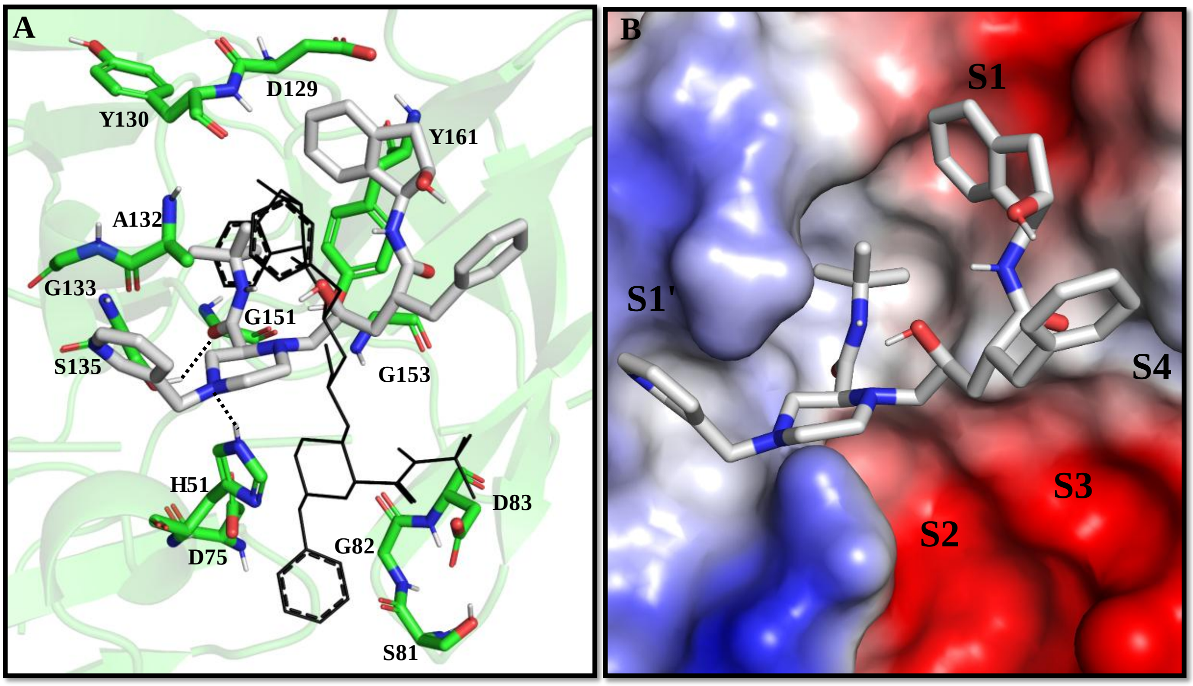

Fig. S4: Average MD-structure of ZIKV protease-Indinavir complex. (A) Interaction of Indinavir with different residues of the ZIKV protease and (B) Placement of Indinavir in the electrostatic surface of the protease.

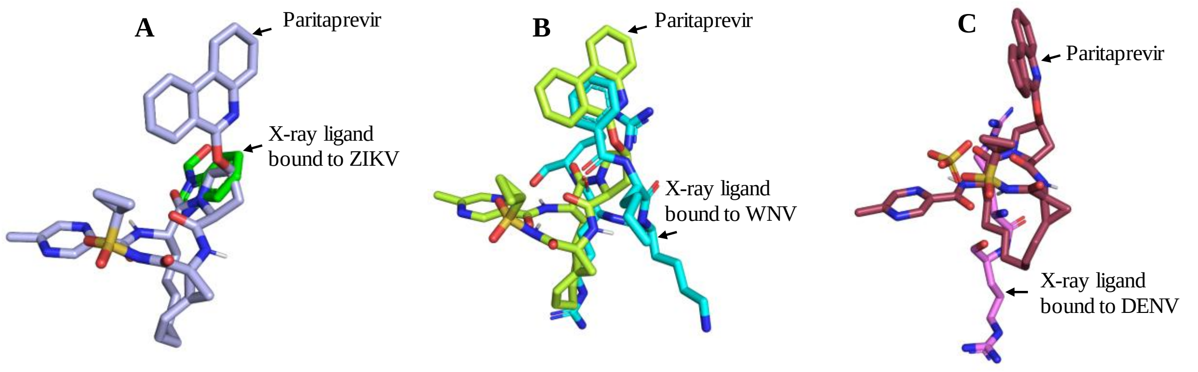

Fig. S5: Comparison of binding modes of Paritaprevir with those of different ligands bound to (A) ZIKV (PDB ID 5H4I), (B) WNV (PDB ID 2PF7), and (C) DENV (PDB ID 3UI1).
